# Comparison of immunohistochemistry with PCR for assessment of ER, PR, and Ki-67 and prediction of pathological complete response in breast cancer

**DOI:** 10.1186/s12885-017-3111-1

**Published:** 2017-02-13

**Authors:** Hans-Peter Sinn, Andreas Schneeweiss, Marius Keller, Kornelia Schlombs, Mark Laible, Julia Seitz, Sotirios Lakis, Elke Veltrup, Peter Altevogt, Sebastian Eidt, Ralph M. Wirtz, Frederik Marmé

**Affiliations:** 1 Institute of Pathology, University of Heidelberg, Im Neuenheimer Feld 220-221, 69120 Heidelberg, Germany; 2 National Center for Tumor Diseases, University-Hospital Heidelberg, Im Neuenheimer Feld 460, 69120 Heidelberg, Germany; 3 German Cancer Research Center, Im Neuenheimer Feld 280, 69120 Heidelberg, Germany; 4 STRATIFYER Molecular Pathology GmbH, Werthmannstr. 1c, 50935 Köln, Germany; 5 Department of Pathology, St. Elisabeth-Krankenhaus, Werthmannstr. 1c, 50935 Köln, Germany; 6 BioNTech Diagnostics GmbH, 55131 Mainz, Germany

**Keywords:** Image analysis, Breast cancer, Ki67, mRNA, RT-qPCR, Prediction, Pathologic complete response, neoadjuvant, Immunohistochemistry (IHC), MammaTyper®

## Abstract

**Background:**

Proliferation may predict response to neoadjuvant therapy of breast cancer and is commonly assessed by manual scoring of slides stained by immunohistochemistry (IHC) for Ki-67 similar to ER and PgR. This method carries significant intra- and inter-observer variability. Automatic scoring of Ki-67 with digital image analysis (qIHC) or assessment of MKI67 gene expression with RT-qPCR may improve diagnostic accuracy.

**Methods:**

Ki-67 IHC visual assessment was compared to the IHC nuclear tool (AperioTM) on core biopsies from a randomized neoadjuvant clinical trial. Expression of ESR1, PGR and MKI67 by RT-qPCR was performed on RNA extracted from the same formalin-fixed paraffin-embedded tissue. Concordance between the three methods (vIHC, qIHC and RT-qPCR) was assessed for all 3 markers. The potential of Ki-67 IHC and RT-qPCR to predict pathological complete response (pCR) was evaluated using ROC analysis and non-parametric Mann-Whitney Test.

**Results:**

Correlation between methods (qIHC versus RT-qPCR) was high for ER and PgR (spearman´s *r* = 0.82, *p* < 0.0001 and *r* = 0.86, *p* < 0.0001, respectively) resulting in high levels of concordance using predefined cut-offs. When comparing qIHC of ER and PgR with RT-qPCR of ESR1 and PGR the overall agreement was 96.6 and 91.4%, respectively, while overall agreement of visual IHC with RT-qPCR was slightly lower for ER/ESR1 and PR/PGR (91.2 and 92.9%, respectively). In contrast, only a moderate correlation was observed between qIHC and RT-qPCR continuous data for Ki-67/MKI67 (Spearman’s *r* = 0.50, *p* = 0.0001). Up to now no predictive cut-off for Ki-67 assessment by IHC has been established to predict response to neoadjuvant chemotherapy. Setting the desired sensitivity at 100%, specificity for the prediction of pCR (ypT0ypN0) was significantly higher for mRNA than for protein (68.9% vs. 22.2%). Moreover, the proliferation levels in patients achieving a pCR versus not differed significantly using MKI67 RNA expression (Mann-Whitney *p* = 0.002), but not with qIHC of Ki-67 (Mann-Whitney *p* = 0.097) or vIHC of Ki-67 (*p* = 0.131).

**Conclusion:**

Digital image analysis can successfully be implemented for assessing ER, PR and Ki-67. IHC for ER and PR reveals high concordance with RT-qPCR. However, RT-qPCR displays a broader dynamic range and higher sensitivity than IHC. Moreover, correlation between Ki-67 qIHC and RT-qPCR is only moderate and RT-qPCR with MammaTyper® outperforms qIHC in predicting pCR. Both methods yield improvements to error-prone manual scoring of Ki-67. However, RT-qPCR was significantly more specific.

**Electronic supplementary material:**

The online version of this article (doi:10.1186/s12885-017-3111-1) contains supplementary material, which is available to authorized users.

## Background

The proliferative activity of individual cells is a hallmark of tumor biological aggressiveness and a key determinant of sensitivity to (neo)adjuvant chemotherapy, thus being among the principal factors guiding clinical management in primary breast cancer [1, 2]. The most widely used method to assess proliferation as well as hormone receptor expression is immunohistochemistry (IHC).

Nuclear staining of the nuclear antigen Ki-67 is most widely used as a surrogate for proliferative activity. Ki-67 is present in the cell nucleus throughout all stages of the cell-cycle excluding the resting phase G0 [3]. The recently proposed St Gallen recommendations for the identification of the intrinsic subtypes using surrogate pathologic-based definitions have underlined the value of Ki-67 as a clinical tool in routine clinical practice [2]. Ki-67 is recommended as a valuable factor to distinguish between Luminal A- and B-like tumors, a fundamental distinction in clinical decision-making today. [4–6].

Despite its widespread use, driven by the premise of solving delicate therapeutic dilemmas combined with several advantages such as universal accessibility, easy application and low cost, the assessment of Ki-67, ER and PR is affected by technical and observer-based variabilities of the IHC method [7, 8]. This can be illustrated by observations, that tumors with as little as 1% positive nuclei still respond to anti-hormonal treatment, which indicates that tumor cells lacking nuclear ER staining within in these tumors do have some extend of ER expression rendering them sensitive to estrogen deprivation or estrogen receptor blockade [9]. While the clinical role particularly of ER testing by IHC is well established, the clinical utility of Ki-67 is still controversial [10]. The reason lies in a series of analytical and preanalytical factors, but also in staining interpretation and scoring [11]. Importantly, attempts to reduce the high discordance rates either by means of formal counting quantification methods (as opposed to simple eyeballing) or by training of individuals have not been successful [12].

Despite methodological concerns, overall a strong correlation of Ki-67 with breast cancer outcome is sufficiently supported, particularly by data originating from randomized clinical trials with central review of biomarkers [10]. This has also been shown in the neoadjuvant setting, where higher Ki-67 values are consistently associated with higher rates of pathological complete response (pCR) [13], a finding which reflects the fundamental link between tumor replication fraction and activity of cytotoxic agents. Still it remains difficult to identify a reasonable cut-off to predict pCR [14].

Two techniques which could circumvent the inter- or intra-observer variability of Ki-67 manual microscopic assessment are automated image analysis and reverse transcription quantitative real-time PCR (RT-qPCR). A trained human eye may achieve an excellent understanding of images and patterns, but is less accurate when it comes to quantification. Computer-based vision methods could represent a solution to this problem by offering standardized image processing and reliable quantification [15]. However with regard to the assessment of tumor proliferation, the areas of interest and staining intensities have to be defined, and measured reproducibly. Also, it is still under debate how to deal with areas of increased proliferative activity (hot spots), and if low intensity staining should be taken into account [16]. Therefore, automated estimations of Ki-67 are highly correlated with manual assessments, but it is not yet certain whether or not they can improve prediction and prognostication [17–19].

RT-qPCR has a series of widely acknowledged methodological advantages over IHC, which appear particularly beneficial in the context of reducing the bias of routine Ki67 assessment; it is quantitative by nature with much wider dynamic range, it does not require an experienced eye, and results are not affected by subjective interpretations [20]. Moreover, access to standardized protocols and automation ensures accurate performance and fast turn-around. In recent years highly specific and sensitive techniques have been developed, which allow for fast and efficient extraction of high-quality nucleic acids from FFPE overcoming the challenges posed by fixation and embedding [21].

Validation of the various available methods for the assessment of Ki-67 requires comparative testing preferably in a specifically defined clinical context. In the present study we used the neoadjuvant setting of a phase II trial randomizing patients receiving anthracyclin/taxane based standard treatment between pemetrexed and cyclophospamide in order to directly compare the assessment of Ki-67 with automatic, quantitative read-out of IHC (qIHC) and the determination of tumor MKI67 mRNA with RT-qPCR on FFPE tissue extracted RNA.

## Methods

### Study population

Core needle biopsies from 101 out of 105 patients (96,2%) with primary invasive breast cancer, that had been enrolled in the H3E-MC-S080 (NCT00149214, Sponsor: Eli Lilly and Company) neoadjuvant phase II study [1], were obtained. All patients had been diagnosed with operable (T2-T4/N0-2/M0) breast cancer at a single institution (National Center for Tumor Diseases, University-Hospital, Heidelberg) had been randomized to receive sequential anthracycline/taxane-based regimens containing either pemetrexed or cyclophosphamide in combination with epirubicin. A written informed consent for the research use of patient biological material was granted at the time of enrolment. The study was approved by the local ethics committee. Complete molecular data (including RT-qPCR data) and clinical follow-up information were available in 83 out of 105 (79%) patients (statistics data set #1). Ki-67, ER, and PR IHC slides were available in 54 (51%) patients for quantitative IHC (statistics data set #2).

### Isolation of tumor RNA

For RNA extraction from FFPE tissue, a single 10 μm curl was processed according to a commercially available bead-based extraction method (RNXtract® kit; BioNTech Diagnostics GmbH, Mainz, Germany). In brief, a lysis buffer was used to liquefy FFPE tissue slices while melting of paraffin was carried out in a thermo-mixer. Tissue lysis was accomplished with a proteinase K solution. Thereafter, lysates were admixed with germanium-coated magnetic particles in the presence of special buffers, which promote the binding of nucleic acids. Purification was carried out by means of consecutive cycles of mixing, magnetization, centrifugation and removal of contaminants. RNA was eluated with 100 μl elution buffer and RNA eluates were then stored at −80 °C until use.

### Gene expression by RT-qPCR

The MammaTyper® is a molecular in vitro diagnostic tool for the assessment of the gene expression levels of the four cancer biomarkers that are required for the clinical management of breast cancer patients in daily routine clinical practice. Instead of using IHC to assess protein expression of HER2, ERα, PR, and Ki-67, with MammaTyper®, it is possible to measure the mRNA transcripts of the corresponding genes (ERBB2, ESR1, PGR, and MKI67), doing so by using routine FFPE material and by achieving accurate, reproducible and objective results. The gene expression data may be then integrated so as to assign individual samples to a molecular subtype of breast cancer.

The mRNA expression levels of ERBB2, ESR1, PGR, and MKI67 as well as of two reference genes (REF), namely B2M and CALM2, were determined by RT-qPCR, which involves reverse transcription of RNA and subsequent amplification of cDNA executed successively as a 1-step reaction. In MammaTyper®, the 6 assays (assay = primer pair and probe specific for the respective target sequence) are duplexed into three assay mixes, each using a pair of hydrolysis probes labelled with different fluorophores for separate detection of the duplexed assays [22].

Each patient sample or control was analyzed with each assay mix in triplicates. The experiments were run on a Versant kPCR Molecular System (Siemens Healthcare, Erlangen, Germany) according to the following protocol: 5 min at 50 ° C, 20 sec at 95 ° C followed by 40 cycles of 15 sec at 95 ° C and 60 s at 60 ° C and according to MammaTyper® instructions for use 140603-90020-EU Rev 2.0.

Forty amplification cycles were applied and the cycle quantification threshold (Cq) values of MKI67 and the two REF genes for each sample (S) were estimated as the median of the triplicate measurements. These were then normalized against the mean expression of the REF genes and set off against a calibrator (PC), to correct for inter-run variations (ΔΔCq method) (Livak et al. 2001). The final values were generated by subtracting ΔΔCq from the total number of cycles to ensure that normalized gene expression obtained by the test is proportional to the corresponding mRNA expression levels, a method that facilitates interpretation of data and clinicopathological correlations. The various calculation steps are summarized in the following formula:

$$ 40\hbox{-} \Delta \Delta \mathrm{Cq}\left(\mathrm{MKI}67\right)\mathrm{S} = 40\hbox{-} \left(\left(\mathrm{Cq}\left[\mathrm{MKI}67\right]\mathrm{S}\ \hbox{--}\ \mathrm{meanCq}\left[\mathrm{REF}\right]\mathrm{S}\right)\ \hbox{--}\ \left(\mathrm{Cq}\left[\mathrm{MKI}67\right]\mathrm{pc}\ \hbox{--}\ \mathrm{meanCq}\left[\mathrm{REF}\right]\mathrm{pc}\right)\right) $$

In 18 patients the MammaTyper® assay failed, because the required amount of RNA was not sufficient for analysis according to pre-specified criteria as described in the instructions for use.

### Pathology and Immunohistochemistry

Tumor grading, tumor typing and immunohistochemistry (ER, PR, Ki-67) was performed on the pretreatment core biopsies on all patients. Pathological complete response (pCR) was determined on tumor resection specimens after completion of neoadjuvant chemotherapy, and was defined as no evidence of residual invasive and ductal disease in the breast and lymph nodes (ypT0,ypN0).

Immunohistochemistry was performed according to previously standardized protocols on an automated IHC platform (Dako Techmate 500) with citrate buffer for antigen retrieval [23] and observing the ASCO/CAP guidelines for immunohistochemistry [7]. The following primary antibodies and corresponding dilutions were used (DakoCytomation, Glostrup, Denmark): ER (clone 1D5, 1:100), PR (clone PgR636, 1:100) and Ki-67 (MIB-1, 1:200). Slides were assessed by quantitative image analysis (qIHC) using the Aperio Image Analysis toolbox (Leica Biosystems, Nussloch, Germany). Staining intensity and percentage of positive nuclei were recorded after manually segmenting tumor from adjacent stroma. Tumors with ER/PR Remmele scores greater than 3 or positive nuclei greater than 1% were considered hormone receptor positive.

### Statistical methods

The Spearman correlation coefficient r was used as a measure of the strength and direction of the linear relationship between variables. 2×2 contingency tables were used to calculate positive percent agreement (PPA) and negative percent agreement (NPA) as a measure of agreement: PPA = 100% x a/(a + c), NPA = 100% x d/(b + d). Receiver Operating Characteristics (ROC) analysis was performed to determine the optimal cut-off for MammaTyper® gene and qIHC protein measurements with pCR as the endpoint. ROC analysis instead of comparing odds ratios to take into account the ratios of clinically relevant false positive and false negative determinations and to identify cut points for each method at clinically relevant prerequisites (i.e., detect all responding tumours). ROC analysis has been used to objectively address each method providing different result codings in a non-parametric manner [24]. On the other hand ROC analysis bears the risk of misinterpreting clinical validity when analyzing heterogeneous populations [25]. However, we have analyzed the response to neoadjuvant chemotherapy within controlled, randomized phase II trial which has defined inclusion and exclusion criteria to have the most comparable basic risk situation. As optimal cut-off for the identification of complete response by the methodologies the point of highest sensitivity still retaining 100% specificity was chosen. The p value reported for evaluating the ROC curve tests the null hypothesis that the area under the curve really equals 0.50 as provided by the statistical program used (GraphPad Prism). The non-parametric Mann-Whitney test was used to confirm the statistical significance when comparing responding versus non-responding tumors and box plots were used to illustrate each case of responding and non-responding tumor above and below the cut-off value. Statistical analyses were performed with JMP SAS (SAS Institute, Cary, NC, USA) and Graph Pad Prism software (Version 5.04; Graph Pad Software Inc., La Jolla, CA, USA).

## Results

### Patient population

**Fig. 1:**
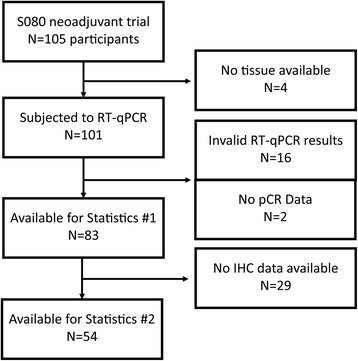
Remark diagram of sample selection

Biopsy tissue was available from 101 out of 105 patients. Gene expression analysis by MammaTyper® was successful in 83 biopsy specimens with full clinical data out of a total of 105 trial participants (Fig. 1). 12 patients out of this group had achieved complete pathological remission (pCR). Basic clinicopathological characteristics of statistics data set #2 that includes quantitative IHC data is listed in Table 1.

**Table 1:** Basic tumor characteristics

	no-pCR		pCR		
Histological Type					*p*= 0.39
Invasive ductal NOS	37	82.2%	9	100.0%	
Invasive lobular	7	15.6%	0	0.0%	
Other	1	2.2%	0	0.0%	
Histological Grade					*p*= 1.00
Grade 2	15	33.3%	3	33.3%	
Grade 3	30	66.7%	6	66.7%	
ypT Category					*p*= 0.81
ypT0	0	0.0%	9	100.0%	
ypT1	27	50.0%	0	0.0%	
ypT2	9	20.0%	0	0.0%	
ypT3	8	17.8%	0	0.0%	
ypT4	1	2.0%	0	0.0%	
ypN Category					*p*= 0.81
ypN0	27	50.0%	9	100.0%	
ypN+	25	46.3%	0	0.0%	
ypNX	2	3.7%			
HER2 status					*p*= 0.68
Neg	36	66.7%	7	13.0%	
Pos	7	13.0%	2	3.7%	

### Comparison of IHC with RT-qPCR based assessment of ER, PR and Ki-67

**Fig. 2:**
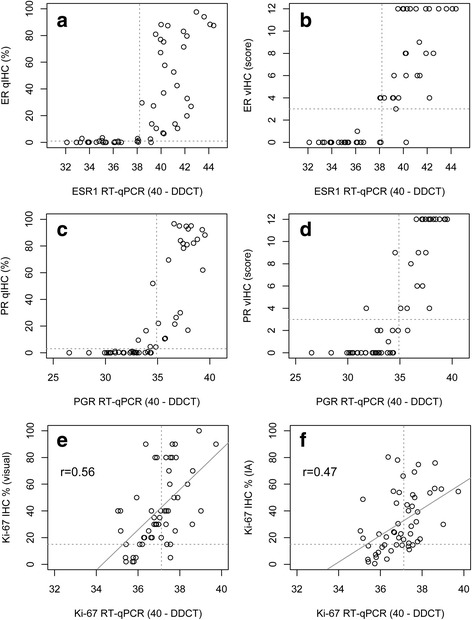
Correlation of RT-qPCR for *ESR1*, *PGR* and *MKI67* with quantitative IHC by image analysis (**a**, **c**, **e**) and visual IHC assessment (**b**, **d**, **f**)

Comparing qIHC of ER with RT-qPCR of ESR1 demonstrated a good overall agreement of 96,6% (PPA 100%; NPA 92.3%) as well as a good correlation looking at the continuous data (spearman’s *r* = 0.82, *p* < 0.0001). Correlation between vIHC of ER and RT-qPCR for ESR1 (spearman’s *r* = 0.85, *p* < 0.0001) and between vIHC and qIHC ER (spearman’s *r* = 0.88, *p* < 0.0001) was high, too. Overall agreement for PR protein and PGR mRNA expression was 91.4% (PPA 83.3%; NPA 100%) comparing qIHC and RT-qPCR and there was a high correlation for the continuous data (*r* = 0.86, *p* < 0.0001). Correlation between vIHC and RT-qPCR and between vIHC and qIHC was very high, too (*r* = 0.88, *p* < 0.0001 and *r* = 0.90, *p* < 0.0001, respectively). Concordance when comparing visual IHC protein with RT-qPCR RNA expression was good for ESR1 as well as for PGR, although slightly lower compared to the agreement between qIHC and RT-qPCR (OPA 91.2%; PPA 90.9%; NPA 91.7%) and for PGR (OPA 92.9%; PPA 88.0%; NPA 96.9%, respectively). For both, ESR1 and PGR, only 4 cases were discordant, with 3 cases each positive by vIHC and negative by RT-qPCR, while 1 case was negative by vIHC and positive by RT-qPCR (Fig. 2). However, several of these discrepancies could be resolved by using quantitative IHC, as these cases were also discrepant when comparing vIHC with qIHC. Moreover, qIHC could delineate quantitative differences of hormone receptor expression at the highest Remmele Score value of 12, where vIHC could not resolve expression differences. In addition, at the lower range of expression levels RT-qPCR based assessment could still determine substantial differences of mRNA levels while the IHC based assessment could not detect any protein expression. The inter-gene spearman correlation was moderate for ESR1 and PGR (*r* = 0.59, *p* < 0.0001), while Ki-67 correlated negatively with PGR (−0.37, *p* = 0.007). While the correlation for ESR1 and PGR protein and RNA expression was high when comparing IHC with RT-qPCR results (spearman’s *r* = 0.82, *p* < 0.0001 and *r* = 0.86, *p* < 0.0001, respectively), the correlation between MKI67 protein and RNA expression was only moderate (spearman’s *r* = 0.56 for vIHC and *r* = 0.47 for qIHC). In contrast the correlation between both methods for Ki-67 assessment by IHC was high (*r* = 0.80, *p* < 0.0001). The median value of Ki-67 proliferation index by image analysis IHC (qIHC) was 23.4%, by conventional visual IHC (vIHC) 35.0%, and 37.01 for RT-qPCR, clearly reflecting the inclusion criteria of the S080 trial which targeted clinically higher-risk patients. Scatter plot analysis displays the positive correlation between RT-qPCR and visual as well as quantitative IHC assessment in Fig. 4.

### Prediction of pCR

**Fig. 3:**
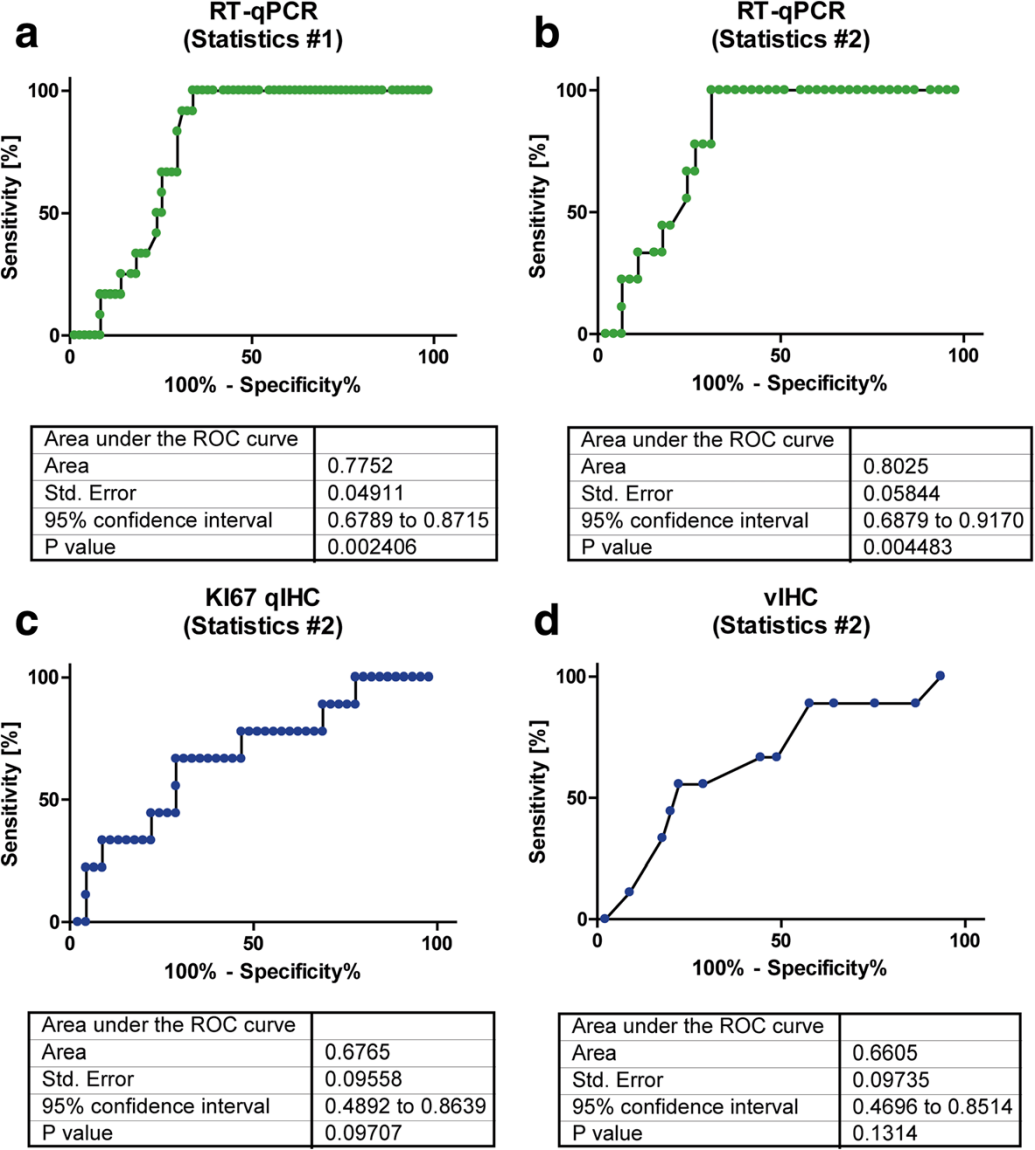
ROC analysis for prediction of pathological complete response by quantifying *MKI67/Ki-67 expression* by RT-qPCR (**a**, **b**) and IHC (**c**, **d**) showing overall increased ability of mRNA assessment to correctly identify responders versus non-responders

**Fig. 4:**
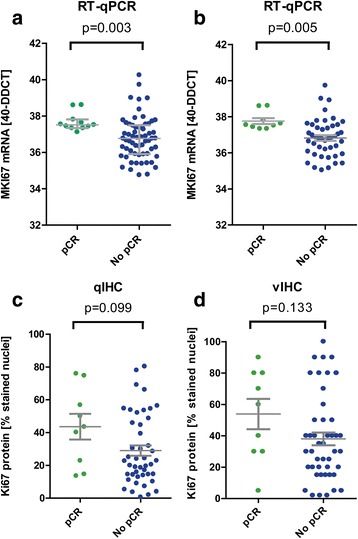
Scatter plots illustrating the distribution of RT-qPCR mRNA (*upper panel*) and qIHC (*lower panel*) and vIHC (*right panel*) protein measurements in relation to the groups of responders (*green dots*) and non-responders (*blue dots*). Differences were tested with the Mann-Whitney test (**a** = data set 1, *n* = 83, **b**, **c**, **d** = common data set, *n* = 54)

To compare the clinical utility we performed a ROC analysis to determine the optimal cutoff for predicting the pCR. The results of the ROC analysis are presented in the graphical plots of Fig. 3. With RT-qPCR, 100% of responders could be detected with a specificity of 68.9% at a 40-ddCT level of 37.31 which almost reflected the median mRNA expression in this cohort (Fig. 3a, b). Conversely, no responder was below RT-qPCR of 37.31 (Fig. 4a). For IHC assessment, it was difficult to determine a reliable cutoff reaching high sensitivity and specificity. With RT-qPCR the area under the curve was 0.78 for the overall cohort and 0.80 for the IHC cohort (Statistics #2) (*p* = 0.002 and *p* = 0.004). For both IHC methods, the ROC was not significant.

Since maximum sensitivity was a pre-requisite, the two methods were compared with respect to specificity, which was found to be substantially higher for MammaTyper® (68.9%) compared to qIHC (22.2%). Using the cut-offs indicated by ROC analysis (37.31 for RT-qPCR, 13.2% for qIHC and 3.5% for vIHC), tumors were characterized as bearing either high or low MKI67 RNA or Ki-67 protein expression, respectively. However, as illustrated in Fig. 4, statistically significant differences between groups were found for the RT-qPCR but not for IHC methods when patients were stratified according to proliferation and pCR (Mann–Whitney *p* = 0.003 and *p* = 0,005 for RT-qPCR and *p* = 0.099 for qIHC and *p* = 0.133 for vIHC). Using the cut-offs obtained by ROC analysis, pCR was observed in 9 of 24 patients (37.5%, *p* < 0.001) with high MKI67 RNA expression but in no patient with low RNA expression. Accordingly for qIHC, pCR was observed in 9 of 44 patients (20.5%, *p* = 0.27) with high Ki-67 labelling index and similarly it was entirely lacking in patients with low proliferation. For RT-qPCR the ROC analysis was also highly significant when only luminal tumors had been assessed, though the sample size was small in this subset (data not shown).

## Discussion

In this study we have validated clinical performance of hormone receptor gene expression by RT-qPCR by comparing predefined cut-offs in a blinded fashion with the current standard of IHC. Furthermore, we have investigated the diagnostic performance of two methods for assessing MKI67 gene expression, namely IHC with computerized quantification of protein and RT-qPCR RNA quantification with the MammaTyper® IVD kit in the setting of pCR prediction. When continuous data were dichotomized to reflect high- and low-MKI67 categories with cut-offs obtained by ROC curve analysis after considering 100% sensitivity, RT-qPCR was significantly more specific than qIHC.

To the best of our knowledge this is the first direct comparison of this kind in the context of a clinical trial. For the mRNA estimation we used the MammaTyper®, a novel in vitro diagnostic test for breast cancer molecular subtyping. To prove the clinical utility of mRNA based assessment, we compared RT-qPCR with conventional visual assessment as well as digital image analysis based determination at a reference pathology lab in the context of a clinical trial. Moreover, the methods were examined with respect to their ability to predict pCR according to Ki-67 protein or MKI67 mRNA expression levels measured on pretreatment core biopsies. Our results indicate, that, when using RT-qPCR valid cut-offs for mRNA expression, which reliably distinguish between non-responding and responding tumors as determined by pCR (ypT0 ypN0) can be identified.

Pathological complete response has gained wide acceptance as one of the strongest predictors of prolonged survival in the setting of neoadjuvant chemotherapy [26, 27]. Therefore, laboratory assays that can efficiently predict a patient’s response to a given preoperative chemotherapeutic combination may serve as tools for individualizing treatment and improving long-term outcomes [28]. As with adjuvant chemotherapy, neoadjuvant regimens also suffer from the fact that substantial therapeutic benefit is restricted only to a fraction of those treated, whereas all patients will experience adverse events because of toxicity [29].

In several neoadjuvant studies Ki-67 protein expression has been investigated in pre-operative biopsies in relation to the response to treatment and in most cases a high Ki-67 proliferation rate was predictive of higher probability of pCR [13]. Fasching et al. analyzed Ki-67 by conventional IHC in core biopsies from 552 patients from a single German institution and showed that a pre-defined 13% cut-off could predict pCR with 94% sensitivity and 36% specificity [30]. Interestingly, our ROC analysis for qIHC requiring 100% sensitivity with the least possible loss on specificity led to an identical cut-off (13.2%) for Ki-67. However, this finding requires careful interpretation, due to differences characterizing the clinical settings between the two neoadjuvant studies and the original work by Cheang [31]. In the latter case, the Ki-67 cut-off was fine-tuned against gene expression profiling in order to distinguish Luminal A from Luminal B tumors in a population containing both high- and low-risk breast cancers, whereas in the neoadjuvant setting the same cut-off was intended to identify the majority of, mainly high-risk, patients that would most likely benefit from preoperative cytotoxic therapy.

Alike what has been repeatedly shown in the adjuvant setting, it appears that the molecular architecture of tumors as defined by the expression of hormone receptors and HER2/neu may act as a modifier of the association between Ki-67 and response to neoadjuvant treatment and between pCR and long-term outcomes [14, 32]. While 101 tumors were available for analysis, the inclusion of 83 or 54 tumors in this study was not based on a statistical rational but was dictated by the availability of tumor tissue with complete RT-qPCR and qIHC data.

A novel aspect of the present work is the comparison between protein-based and mRNA-based methods for the assessment of tumor proliferation. Our findings highlight the feasibility of using RT-qPCR for the routine assessment of ESR1, PGR & MKI67 in order to assist the selection of breast cancer patients for neoadjuvant treatment. Even though both RT-qPCR and qIHC of MKI67/Ki-67 could be calibrated to maximize negative predictive value, only with the former this was achieved whilst ensuring sufficient specificity, which if validated would signify that MammaTyper® could help a considerable number of patients safely forego unnecessary treatment. These data collectively indicate that MammaTyper®MKI67 RNA was overall more representative of the true proliferation state of the tumor than was computer assisted Ki-67 protein estimation, a finding that is worth validating in larger datasets.

Significant correlations between conventional Ki-67 visual assessment and RT-qPCR have been previously reported [33, 34], indicating a strong biological link between mRNA and protein expression despite methodological variations, as is further indicated by comparable prognostic hazard ratios obtained by both methods [35]. To the best of our knowledge however, our study is the first to compare image analysis with RT-qPCR for the assessment of tumor proliferation with the additional advantage of using material from a randomized clinical trial. The correlation between mRNA and protein was significant but moderate, a finding which may reflect post translational modifications or may be related to the increased dynamic range of RT-qPCR as compared to IHC. Another possible explanation might be that mRNA levels are a reflection of the average gene expression in the entire FFPE slice, whereas IHC may be biased in favor of selected “representative” tumor areas. Even in the case of image analysis systems, inspection of digitalized images and manual identification of tumor areas is necessary before automatic scoring.

Computerized methods have been recommended as a solution to the problem of subjectivity in the visual assessment and scoring of IHC-stained slides. Not surprisingly, Ki-67 scores from image analysis systems are generally in close agreement with those of manual methods because manual scoring for research purposes is customarily performed by a pathologist with longstanding experience in the field [17, 36]. It is worth mentioning, however, that in a routine decentralized setting, digital processing and scoring of slides would probably outperform manual assessment which is prone to considerable subjectivity often not improved upon standardization [37]. Digital analysis yields more reproducible results with regard to staining intensity, by facilitation the definition of low grade staining intensities. Definitive conclusions would require comparisons between all three methods (central versus local versus automatic) performed preferably in the prospective retrospective setting of a large multi-center trial.

Multi-gene molecular signatures have also been tested as a way for predicting pCR in patients with breast cancer [38–40]. However, generalized use of these commercialized assays is limited by their increased cost and the requirement to run in centralized platforms or both. Interestingly, proliferation genes, including MKI67, are often heavily weighted in multi-gene scores which serve as estimators of a patients’ risk of developing recurrences. This is perhaps one of the reasons why multi-gene tests do not always prove to be convincingly superior to conventional or less sophisticated methods for tumor risk stratification [35, 41, 42], leading some authors to question their cost-effectiveness [43]. Moreover, for several commercially available tests, neither doctors nor consumers can gain access to the continuous expression data of individual proliferation markers that make up the final risk scores. This restriction overall minimizes the possibility of potentially interesting comparisons between proliferation motifs or scores and single proliferation markers based on RT-qPCR or IHC. Strikingly, our ROC curve analysis of MKI67 40-ΔΔCq values for the prediction of pCR displayed performance characteristics that are comparable with those of a 50-gene predictor of tumor recurrence risk developed by supervised training of Cox models [39]. Along these lines, single-gene MKI67 RT-qPCR may be worth considering as a golden means for assessing tumor proliferation due to its unique ability to combine technical advancements and diagnostic accuracy with more affordable pricing.

## Conclusions

Image analysis-assisted scoring of ER, PR and Ki-67 IHC and quantification of ESR1, PGR and MIKI67 RNA expression with RT-qPCR both represent promising alternatives to conventional visual estimation and may assist in improving reproducibility and accuracy in the field. However, RT-qPCR assessment of tumor proliferation was overall more accurate than quantitative IHC. This is the first study to compare tumor MKI67 gene expression by RNA and protein assessment in a prospective retrospective neoadjuvant setting. Due to the relatively small sample size, these data should be considered preliminary and worth validating in larger datasets.

## Additional file

Additional file 1: Raw data; qPCR data, quantitative and visual IHC values, pathologic complete response yes/no. (XLSX 42 kb)

## Abbreviations

CISH: Chromogenic in situ hybridization
Cq: Quantification cycle
DDFS: Distant disease free survival
ESR1/ER: Oestrogen receptor alpha; ERBB2/HER2
FEC: Fluorouracil & epirubicin, cyclophosphamide chemotherapy
FFPE: Formalin fixed paraffin embedded
GOI: Gene of interest
HR: Hazard ratio
IHC: Immunohistochemistry
MKI67/Ki67: marker of proliferation Ki-67
mRNA: Messenger ribonucleic acid
NPA: Negative percentage agreement
OPA: Overall percentage agreement
OS: Overall survival
PGR/PgR: Progesterone receptor
PPA: Positive percentage agreement
REF: Reference gene
RT-qPCR: Reverse transcription quantitative real time polymerase chain reaction
